# Phytochemicals and Biogenic Metallic Nanoparticles as Anticancer Agents

**DOI:** 10.1155/2016/3685671

**Published:** 2016-02-23

**Authors:** Pasupuleti Visweswara Rao, Devi Nallappan, Kondeti Madhavi, Shafiqur Rahman, Lim Jun Wei, Siew Hua Gan

**Affiliations:** ^1^Biotechnology Program, Faculty of Agro-Based Industry, Universiti Malaysia Kelantan, Campus Jeli, 17600 Jeli, Malaysia; ^2^Department of Biochemistry, Sri Venkateswara Medical College, Tirupati, Andhra Pradesh 517502, India; ^3^Department of Parasitology, Graduate School of Health Sciences, Kobe University, Kobe 654-0142, Japan; ^4^Department of Fundamental and Applied Sciences, Universiti Teknologi Petronas, 32610 Tronoh, Malaysia; ^5^Human Genome Centre, Universiti Sains Malaysia, 16150 Kubang Kerian, Malaysia

## Abstract

Cancer is a leading cause of death worldwide. Several classes of drugs are available to treat different types of cancer. Currently, researchers are paying significant attention to the development of drugs at the nanoscale level to increase their target specificity and to reduce their concentrations. Nanotechnology is a promising and growing field with multiple subdisciplines, such as nanostructures, nanomaterials, and nanoparticles. These materials have gained prominence in science due to their size, shape, and potential efficacy. Nanomedicine is an important field involving the use of various types of nanoparticles to treat cancer and cancerous cells. Synthesis of nanoparticles targeting biological pathways has become tremendously prominent due to the higher efficacy and fewer side effects of nanodrugs compared to other commercial cancer drugs. In this review, different medicinal plants and their active compounds, as well as green-synthesized metallic nanoparticles from medicinal plants, are discussed in relation to their anticancer activities.

## 1. Introduction

Cancer is one of the leading causes of death in the world. According to the 2014 World Cancer Report, approximately 14 million new cancer cases and 8.2 million cancer-related deaths were reported in 2012. Among the different types of cancer, lung cancer is associated with the greatest mortality (1.5 million deaths), followed by liver (745 000 deaths), stomach (723 000 deaths), colorectal (694 000 deaths), breast (521 000 deaths), and esophageal cancer (400 000 deaths) [1]. The number of new cancer cases is expected to increase by 70%, from 14 million to 22 million, in the next 2 decades [2]. The populations of Africa, Asia, and Central and South America represent 70% of all cancer deaths and 60% of the total new annual cancer cases worldwide [3].

Several therapies are available to treat various types of cancer. Chemotherapy in combination with cytotoxic agents is the most commonly utilized therapy to control many types of cancer [4]. Nevertheless, these therapeutic approaches are linked to severe side effects, especially multidrug resistance (MDR) [5, 6]. There are various undesirable side effects of chemotherapy alone or in combination with cytotoxic drug therapy or radiation therapy [7]. Based on these undesirable side effects, the National Cancer Institute (USA) has encouraged the investigation of the potential antitumor activities of plant extracts [8, 9].

Natural compounds isolated from medicinal plants are believed to be promising leads in the development of anticancer drugs. Screening of medicinal plants and their active constituents for various biological activities, such as anticancer activity, has been a major interest since the 1960s [8]. Medicinal plants have shown activity against various metabolic diseases and cancers. However, because of their minimal size, green-synthesized nanoparticles from medicinal plants have become a keen interest of researchers.

Nanoparticles play a critical role in refining the compatibility and bioavailability of natural products in the treatment of several chronic diseases, including cancer. Among the metallic nanoparticles, silver nanoparticles (AgNPs) are a popular choice in disease management because of their specific interaction with and disruption of the mitochondrial respiratory chain. AgNPs disrupt mitochondrial function by inducing the generation of reactive oxygen species and suppressing ATP synthesis, which lead to DNA damage. In this context, the present review focuses on medicinal plants and green-synthesized nanoparticles from medicinal plants with potential anticancer activities and their future applications.

## 2. Role of Phytochemicals in Cancer

Since ancient times, numerous medicinal plants extracts and their active components have been reported to have potential uses as anticancer agents. Numerous studies have reported that medicinal plants display anticancer and cytotoxic activities [10]. Polyphenols, such as phenolic acids, flavonoids, terpenes, and alkaloids, possess the biological potential of medicinal plants [11–13]. Triterpenoids such as ursolic acid, oleanolic acid, boswellic acids, pomolic acid, avicins, oleanolic acids, and fomitellic acids have been reported to exert cytotoxic effects [14]. Furthermore, flavonoids such as kaempherol, myricetin, quercetin, and rutin have been reported to display anticancer properties [12]. Additionally, several alkaloids such as matrine and sanguinarine have been reported to possess anticancer activities [15]. Researchers have demonstrated the possible mechanisms of action of medicinal plants and their active ingredients or active compounds, which may exert these mechanisms individually or in combination with other compounds present in the plants. One major potential mechanism of action for reducing damage caused by disease is antioxidation [16]. Liu reported the various potential activities of phytochemicals in cancer [17]. Another review detailed the biological efficacies, especially the potential activities, of flavonoids against cancer [18]. Numerous phytochemicals present in medicinal plants can induce cytotoxicity against various types of cancerous cells. Some of the plants and their phytochemicals that have been reported to possess anticancer activity are listed in Table 1. In Table 2, the isolated compounds and their anticancer activities are presented.

## 3. Nanotechnology in Cancer Treatment

The field of nanotechnology holds potential to transform cancer diagnostic methods and therapeutic technologies. Advances in materials science and protein engineering have given rise to novel nanoscale targeting approaches that may increase safety and therapeutic efficacy in cancer patients. Nanotechnology involves the application of structures, characterization, design, devices, production, and systems at the nanometer scale. The common challenges associated with existing cancer treatments are localization of the therapy to tumor sites, drug resistance by tumors, and short drug circulation times. In addition, cancer drug toxicity leads to major complications, such as heart problems and low white-blood-cell counts. There are several modes for delivery of nanoparticles to tumors, such as liposome-mediated drug delivery (doxorubicin and daunorubicin), biodegradable and biocompatible polymeric nanoparticle delivery [polycaprolactone (PCL) and poly(lactic-co-glycolic acid) (PLGA)], and drug delivery of dendrimers [(poly-l-lysine)-octa(3-aminopropyl) silsesquioxane] surface-altered with cyclo(RGDFK) [19, 20].

## 4. Green Synthesis of Nanoparticles

**Figure 1:**
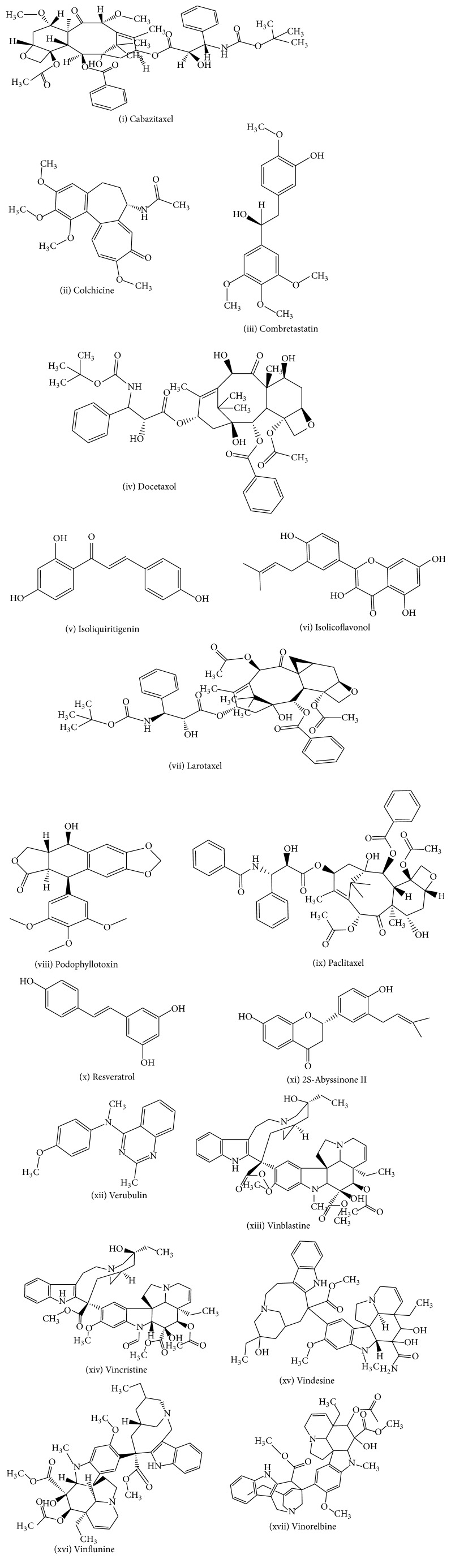
Structures of compounds isolated from medicinal plants used as anticancer agents.

Various classes of alkaloids and flavonoids have been isolated from several medicinal plants and have shown cytotoxic efficacy against numerous types of cancerous cells both* in vitro* and* in vivo*. Some of these compounds exert their cytotoxic effect by inhibiting cancer cell growth (Figure 1).

**Figure 2:**
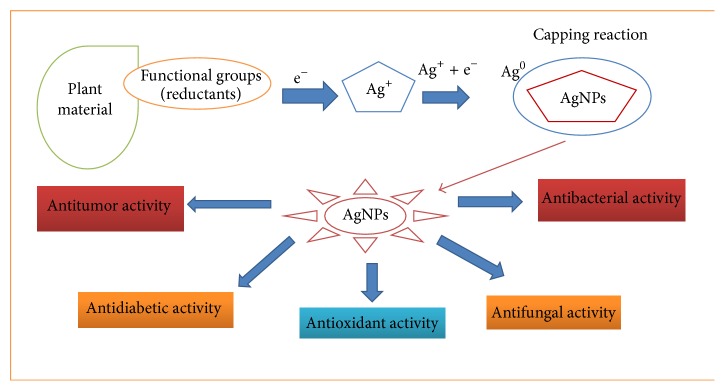
Biogenic synthesis of nanoparticles and their biological activities, including anticancer activity. The figure describes the formation of metallic nanoparticles [silver nanoparticles (AgNPs)] using plant materials. The functional groups in plant materials act as reductants by donating electrons to reduce silver ions in silver nitrate, which leads to the synthesis of AgNPs. Biogenically synthesized AgNPs have several biological efficacies. Other types of metallic nanoparticle formation are not shown in this figure.

Nanotechnology is one of the most prominent and promising fields for the development of new applications in medicine. Unfortunately, there are only a few nano-based products that are currently used in medical applications. To date, researchers have mainly focused on metallic nanoparticles because of their rapid actions. Metallic nanoparticles have been recognized to have unique physical and chemical properties based on their quantum size, which lead to a range of interesting biomedical applications. Silver, gold, zinc oxide, iron oxide, copper oxide, and aluminum oxide are the most commonly used metallic nanoparticles. Silver and gold are among the most important, flexible, reliable, and prominent ions in use in the green synthesis of nanoparticles from medicinal plants and their components. The formation of nanoparticles and their biological efficacies, including anticancer activities, are shown in Figure 2.

## 5. AgNPs

Silver has the highest electrical and thermal conductivity of any metal. AgNPs play an important role in the field of nanotechnology because of their extraordinary properties, including chemical stability, conductivity, catalytic activity, and biological activities such as antibacterial, antifungal, antiviral, and anti-inflammatory activities. Because of their cytotoxic potential, AgNPs have been extensively investigated in cancer research. The biological synthesis of nanoparticles using several components of medicinal plants has become a new trend because of the reduced side effect profiles of the resulting nanoparticles compared to other commercial drugs. Currently, many researchers are focusing on green-synthesized nanoparticles from medicinal plants to investigate various biological efficacies, such as their antimicrobial [21], anticancer [22], antidiabetic [23], and antimalarial [24] properties.

According to Prabhu et al. [25], green-synthesized AgNPs from methanolic extracts of* Vitex negundo* L. showed 50% inhibition of the cell viability of human colon cancer cell lines (HCT 15) when administered at 20 *μ*g/mL. Overall, the concentration, size, and shape of the AgNP are important in their biological efficacy. The increased cytotoxic efficacy of AgNPs at increasing concentrations has also been reported in HeLa cell lines. A recent study of biologically synthesized AgNPs from* Acalypha indica* reported cytotoxic properties against MDA-MB-231 cells, that is, human breast tumor cells [26].

Generally, the cytotoxicity of AgNPs and gold nanoparticles (AuNPs) against cancerous cells tends to increase with their concentration. In 2014, Jeyaraj et al. deduced that (50 *μ*g/mL) AgNPs induced 100% cell death of MCF-7 human breast tumor cells [27]. However, AgNPs derived from mushrooms showed significant cytotoxicity against MDA-MB-231 cell lines at a comparatively low concentration (6 mg/mL) [28]. AgNPs from* Andrographis echioides* inhibited the growth of MCF-7 cells, an extensively used human breast adenocarcinoma cell line, at 31.5 *μ*g/mL [29]. Additionally, biofunctionalized green-synthesized AgNPs exhibited potential cytotoxic activity against HT29 human colon adenocarcinoma cells [30].

AgNPs biosynthesized from* Premna serratifolia* leaves displayed significant anticancer activity in carbon tetrachloride- (CCl_4_-) induced liver cancer in Swiss albino (BALB/c) mice [91]. A recent study by Sre et al. (2015) reported the cytotoxic activity of biologically synthesized AgNPs from* Erythrina indica* on MCF-7 (breast cancer) cells and HepG2 (hepatocellular carcinoma) cells [92]. This study also clearly indicated that the viability of cancerous cells decreases with increasing AgNP concentrations. Another research group reported the biocompatibility of AgNPs with the stem latex of* Euphorbia nivulia*. AgNPs that were synthesized using the latex of* Euphorbia nivulia* exhibited potentially cytotoxic effects against human lung carcinoma (A549) cells in a dose-dependent manner [93].

**Figure 3:**
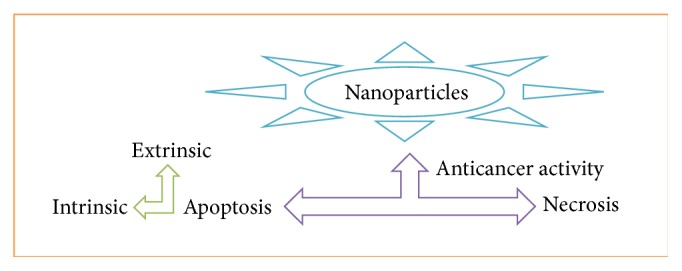
A simplified diagram of anticancer activities triggered by nanoparticles in tumor cells.

**Figure 4:**
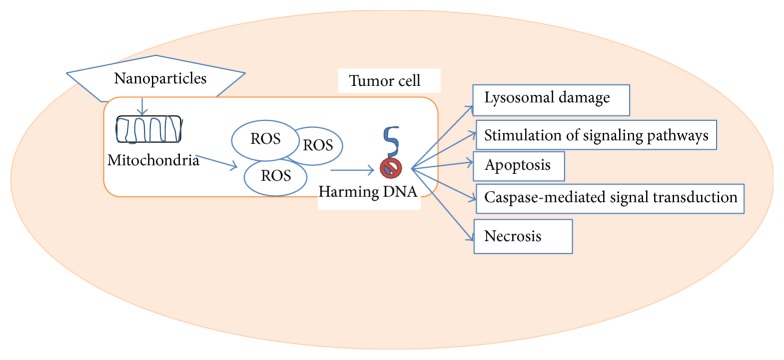
The mechanisms of apoptosis and necrosis mediated by nanoparticles in tumor cells.

*Annona squamosa* seed extract is reported to display good anticancer activities against human hepatoma and breast cancer cells both* in vitro* and* in vivo* [94, 95]. Another study reported the cytotoxic effects of various solvent extracts of the* Annona squamosa* fruit pericarp against Dalton's lymphoma cells and HeLa cells. The chloroform extract of* Annona squamosa* pericarp exerted good cytotoxic effects against different cell lines used in one study [96]. AgNPs synthesized from* Annona squamosa* leaf extracts were also reported to possess potential cytotoxicity against breast cancer (MCF-7) cells. The cytotoxic capacity of metallic nanoparticles against various types of cancer cell lines is mediated by necrosis, stimulation of signaling pathways, lysosomal damage, caspase-mediated signal transduction, and apoptosis (Figures 3 and 4). Several factors, such as particle shape, size, and surface chemistry, influence the cytotoxicity of AgNPs. Apoptosis is characterized by nuclear shrinkage, blebbing, and loss of membrane integrity in dying cells following treatment with AgNPs. The formation of apoptotic nuclei, that is, condensed chromatin structures, was observed in MCF-7 cells treated with AgNPs but not in untreated cells.

Medicinal plants have been used widely in anticancer studies due to their high efficacy and limited side effects. The anticancer efficacy of medicinal plants has been studied in clinical trials and has shown positive results. According to Fleischauer et al. [97],* Allium sativum* (garlic) exerted a protective effect against gastrointestinal cancers based on epidemiologic studies [97]. Administration of garlic enhanced the efficacy of natural killer cells in patients with advanced digestive system cancer [98]. In addition, curcumin, a polyphenol (diferuloylmethane) derived from the rhizome of turmeric (*Curcuma longa Linn*), displays anticancer efficacy through its multiple actions on apoptosis, cell cycle, mutagenesis, metastasis, and oncogene expression [99]. According to a nonrandomized open-label study by Dhillon et al. (2008), curcumin showed positive results in two pancreatic bladder cancer patients by prolonging the disease for more than 18 months and inducing tumor regression [100].

Camptothecin is another natural alkaloid that can be extracted from several plants, such as* Mappia foetida* and* Canzptotheca acirminata*. This compound displays potent antitumor efficacy by targeting topoisomerase I, an enzyme involved in the relaxation of DNA supercoils [101]. A phase 1 clinical trial showed that 20-(S)-camptothecin and 20-(S)-9-nitrocamptothecin exerted significant antitumor effects in patients with breast cancer, prostate cancer, and melanoma. Paclitaxel is a member of the class of taxanes, which are highly hydrophobic molecules with low solubility in water. Albumin-coated paclitaxel (Abraxane) was approved by the FDA in 2005 for metastatic breast cancer treatment and showed good efficacy against advanced pancreatic cancer. Nanospheres of albumin-coated paclitaxel can be used to transport an insoluble drug [102].

The root of ginseng (*Panax ginseng*) is a popular traditional medicine in Asia. Consumption of ginseng before a cancer diagnosis increased the overall survival rate among breast cancer patients [103]. In another randomized placebo-controlled trial,* P. ginseng* consumption enhanced certain aspects of physical and mental functioning in gynecologic or hepatobiliary cancer patients [104]. In addition,* Viscum album*, also known as European mistletoe, is another very frequently suggested cancer therapy. Approximately 23 clinical trials were performed on this plant extract up to 2003, and 19 of these trials showed positive results on quality of life, survival, and tumor suppression in cancer patients [105].

## 6. AuNPs

AuNPs also exhibit special properties, such as surface plasmon resonance (SPR) and the ability to bind to thiol and amine groups, thus permitting surface modifications and use in biomedical applications. The* in vivo* and* in vitro* cytotoxic effects of AuNPs have been reported in several studies, some of which showed that AuNPs exhibit anticancer properties through the induction of oxidative stress [106]. Mechanistically, AuNP-treated HeLa cervical carcinoma cells displayed increased generation of reactive oxygen species, leading to the oxidation of several molecules such as lipids and proteins, and enhanced mitochondrial activity, ultimately leading to the death of the cancerous cells. In addition, exposure to 20 nm AuNPs was reported to cause oxidative stress in MRC-5 fetal human lung fibroblasts.

A recent study reported that AuNPs synthesized from* A. leptopus* exhibit good anticancer activity against MCF-7 breast cancer cells at 257.8 *μ*g/mL [107]. Another study demonstrated the cytotoxic efficacy of* Cassia tora* against colon cancer cell lines. The study revealed that the activity of* C. tora* at three different doses (25, 50, and 75 *μ*g/mL) was dose-dependent; the 75 *μ*g/mL dose showed the highest activity against the colon cancer cell lines [108]. Green-synthesized AuNPs from* Gymnema sylvestre* leaf extracts (*G. sylvestre*) were also investigated for their anticancer effects against hepatocellular carcinoma (HepG2) cells. The study revealed that these AuNPs exerted significant cytotoxic effects against HepG2 cancer cells at a maximal concentration of 250 *μ*g/mL [30]. Another study reported that* Moringa oleifera* flower aqueous extract-synthesized AuNPs showed anticancer activity against A549 lung cancer cells. A dose of 50 *μ*g/mL AuNPs showed potential activity against this lung cancer cell line [109].

## 7. Iron Oxide Nanoparticles

Iron oxide nanoparticles induce antitumor activity directly and indirectly via nontoxic wavelength radiation (near-infrared (NIR), oscillating magnetic fields) that is readily absorbed by toxic stimuli of reactive oxygen species production. The particulate nature of iron oxides enables them to bind covalently to the tumor site. In addition, iron oxide can transform radiant energy into reactive oxygen species, which ultimately reduces the adverse damage to healthy tissues and cells. Spherically shaped iron oxide nanoparticles were endorsed by the EU to be used as an agent to treat prostate cancer and to induce magnetic tumor hyperthermia in the brain in combination with chemotherapy or radiotherapy. Hyperthermic therapy using iron oxide nanoparticles tends to kill tumor cells at a temperature at 150–400°C. Nanomaterials receive energy from external sources, such as magnetic fields and near-infrared (NIR) radiation, and transform it into heat, which can kill the tumor cells [110, 111]. The generation of biologically green-synthesized iron oxide (Fe_3_O_4_) nanoparticles from seaweed (*Sargassum muticum*) has also been reported recently [112].

## 8. Titanium Dioxide Nanoparticles

Titanium oxide is an inorganic nanoparticle that can be surface-engineered to inhibit tumor growth. According to Thevenot et al. (2008), titanium oxide nanoparticles were incorporated into the cytoplasm and the cell membrane of T-24, HeLa, and U937 cancer cells [113]. In another study, NIR light-stimulated titanium dioxide nanoparticles appeared to be more effective than UV-stimulated nanoparticles in inducing antitumor activity in HeLa cells* in vitro* and in BALB/c nude mice* in vivo* [114].

## 9. Cerium Oxide Nanoparticles

Cerium oxide nanoparticles have “smart” capability to specifically inhibit the growth of irradiated cancer cells without harming the surrounding tissue due to oxidative stress and radiation-induced damage. These nanoparticles can selectively induce apoptosis and high levels of oxidative stress in cancer cells without damaging normal tissues. A recent study revealed that cerium oxide nanoparticles potently kill L.3.6pl pancreatic cancer cells while protecting normal cells. A similar result in which cerium oxide nanoparticles showed low inhibitory potential on normal human cell lines compared to cancer cell lines has been reported [115]. In addition, administration of cerium oxide nanoparticles can lead to DNA damage, resulting in tumor cell death. Cerium oxide nanoparticles increase the levels of reactive oxygen species in tumor cells, leading to apoptosis, but do not exert genotoxic effects. The antitumor activity of cerium oxide nanoparticles is greatly dependent on their size and shape, although both small- and large-sized nanoparticles induce DNA damage in tumor cell lines [116].

## 10. Bimetallic Nanoparticles

In addition to single metal nanoparticles, a mixture of different metals, especially two metals (bimetallic), may elicit significant cytotoxic effects against breast cancer cells. Silver–selenium (Ag-Se) bimetallic nanoparticles synthesized using quercetin and gallic acid displayed potential antitumor activity against Dalton's lymphoma cells [117]. Another study revealed that silver–gold bimetallic nanostructures exhibited significant cytotoxic effects against MCF-7 breast cancer cells [118, 119]. A recent study reported the potential cytotoxic efficacy of gold-platinum (Au-Pt) bimetallic nanoparticles in cervical cancer. The gold and platinum ions were efficaciously condensed together at room temperature to produce Au-Pt nanostructures. The findings of that study revealed various approaches for the advancement of extremely valuable bimetallic nanostructures with cytotoxic activities [120].

## 11. Importance of Nanoparticle Size and Shape to Disease Treatment

Current studies deduced that the shape, size, and surface properties of nanoparticles are important for achieving targeted anticancer activity with minimal side effects, as these characteristics influence the circulation time, cellular uptake, biodistribution, and cancer drug delivery of nanoparticles. For instance, nanoparticles with a diameter of less than 100 nm are able to penetrate tumor cells easily through a retention effect and through enhanced vascular permeation. Nanoparticles are predominantly designed to be spherical due to their ease of manufacture [121].

According to van de Ven et al., 400 nm disc-shaped nanoparticles more readily bind to melanoma cells than spheroid nanoparticles in a mouse model [122]. The researchers also reported that the 400 nm discs were less likely to end up in the liver. Moreover, disk-shaped nanoparticles (nanodisks) attached to the tumor surface longer than spherical nanoparticles. This property enhances the efficiency of transfer of therapeutic drugs to the tumor [123]. Another report stated that rod-shaped nanoparticles more effectively delivered chemotherapy drugs to breast cancer cells than spherical nanoparticles [124]. In addition, another group of researchers deduced the first application of needle-like shaped polystyrene or PLGA nanoparticles: the successful penetration of the endothelial cell membrane to deliver siRNA into the cytoplasm [125]. Needle-like shaped nanoparticles induced high gene-expression efficiency, indicating that nanoparticle shape is a key parameter for successful siRNA therapy. Studies showed that nanodiamonds can be bound to chemotherapy drugs to treat brain tumors, as the nanodiamond and chemotherapy drug combination remains in the tumor longer than the chemotherapy drug alone, which should increase its effectiveness [126].

Different sizes of AuNPs promote anticancer activity via distinct mechanisms. For instance, 9 nm or smaller spherically shaped AuNPs could cross the nuclear pore complex of tumor cells, whereas 39 nm AuNPs carrying a nuclear transport signal can be delivered to the nucleus [127].

The anticancer activity of nanoparticles is size-dependent; in general, the smaller the nanoparticles, the greater the inhibition of cancer cell proliferation. Small-sized nanoparticles can penetrate deeply into tumor tissue more effectively and easily than large-sized nanoparticles. Based on previous studies, gold nanoparticles have gained much attention due to their easy fabrication, controllable size and shape, tunable surface functionalization, and good biocompatibility in cancer treatment [128]. Some of the reported nanoparticles with anticancer activity at different sizes and shapes are listed in Table 3.

## 12. Mechanisms of Action of Nanoparticles

Necrosis and apoptosis induce cell death in tumor cells, and these forms of cell death can be quantitatively differentiated via morphology. The nuclear contents and the cytoplasm of necrotic cells appear to leach from the cells, whereas the nuclei of apoptotic cells appear shrunken with heavily condensed chromatin [129]. AgNPs are promising antitumor agents. Based on a report by Govender et al., 2012, biogenically synthesized AgNPs using* Albizia adianthifolia* induced significant apoptosis of 57 ± 0.59% and necrosis of 17 ± 0.79% of human lung carcinoma cells [130]. Apoptosis can be further divided into two pathways, intrinsic and extrinsic. Mitochondria play a vital role in intrinsic apoptosis via the depolarization of the mitochondrial membrane due to mitochondrial permeability transition (PT) pore opening. This process eventually causes a low ATP concentration and induces the intrinsic apoptosis pathway. The extrinsic apoptotic pathway is mediated by the CD95 death receptor, which recruits the adapter protein Fas-associated death domain (FADD). The adapter protein FADD binds to and activates caspase-8 via the formation of a death-inducing signaling complex.

Necrosis occurs as a result of the disruption of cellular and nuclear membranes under extreme physiological conditions. Rupture of the cellular membrane differentiates necrosis from apoptosis. Based on a report by Qi et al., 2005, chitosan nanoparticles induced morphological features of necrosis, such as disruption of the cytoplasm and appearance of remnants of swollen organelles, in MGC803 cells. Administration of chitosan nanoparticles to MGC803 cells caused complete disruption of the plasma membrane, and the contents of the cells leaked out within 24 hours [83].

In addition, AuNPs induced cancer activity via several mechanisms, such as phytothermal ablation, mechanical damage, and delivery of anticancer agents (tumor necrosis factor or doxorubicin), with minimal injury to healthy cells [131]. Zinc oxide nanoparticles induce tumor cell death by NADPH-dependent oxidative burst and apoptotic signaling. In a recent study, four representative ZnONP samples of different size and specific surface area showed a remarkably similar impact on cytotoxicity and DNA fragmentation in macrophages of mice in an ap47phox- and Nrf2-independent manner. ZnONP induced necrosis and apoptosis in these macrophages due to their important role in the regulation of immune responses during inflammation and clearance of inhaled particulates. ZnONP enhanced a rapid induction of nuclear condensation, DNA fragmentation, and the formation of hypodiploid DNA-containing nuclei and apoptotic bodies [132].

In a recent study, carbon-based nanoparticles showed significant antitumor activity on human monocyte-derived macrophages. Both single-walled carbon nanotubes and multiwalled carbon nanotubes induced tumor growth by penetrating the nuclear and plasma membranes. These results were supported by the Neutral Red assay and ultrastructural analysis, which indicated increases in cell death. Carbon nanoparticles induced toxicity to human monocyte-derived macrophages in the concentration range from 0.31 to 10 *μ*g/mL. The proportion of necrotic cells was higher than that of apoptotic cells. Administration of carbon nanoparticles to macrophages induced lipid peroxidation and the internal release of digestive enzymes, which caused cell death. Several other factors, such as reduced membrane integrity, ion exchange, DNA damage, and hampered phagocytosis, could lead to cell death [129].

## 13. Future Prospects of Nanotechnological Approach of Phytochemicals

Currently, various clinical researches focused on the effectiveness of nanoscale phytochemicals on biological systems with more than 20 nanoparticle therapeutics available for various clinical applications. Albumin-bound paclitaxel (Abraxane®, described in patents WO2014105644 and WO2008057562) and liposomal daunorubicin (Daunoxome®, described as patented products EP0004467 and US20070286897) are two examples of successful inventions of natural products formulations based on nanotechnological approach [133].

The advancement of innovative nanotechnological approaches may provide a solution to limitations faced by many of phytochemicals' physicochemical and pharmacokinetics properties. One of the ways is via the implementation of suitable nanorange carriers which may permit a slow, sustained but controlled release of the encapsulated phytochemicals [133]. Other examples include effective delivery of nutrients, rapid sampling of chemical and biological impurities, bioseparation of proteins, and nanoencapsulation of nutraceutical, DNA microarrays, microelectromechanical systems, and microfluidics [134]. Apart from this, combination of nanotechnology and phytochemicals leads to advancement in the field of cosmetics. For example, the incorporation of ZnO and titanium dioxide (TiO_2_) nanoparticles provides higher protection from the sun. Liposome-based* Aloe vera* extracts of less than 200 nm diameter have been confirmed to allow higher proliferation and lead to enhanced collagenase* in vitro* using fibroblast and epidermal keratinocytes [135].

The interrelationship of phytochemicals and nanotechnology technologies brings wide future perspective in the food industry due to the availability of many simpler forms of food-grade lipids, multiple emulsions, and solid lipid nanoparticles. Nanoemulsion-based delivery systems have promised to be a good solution to improve the biological efficacies of different phytochemicals and their oral bioavailabilities. In the same manner, polymer micelles also exhibit potential to enhance water dispersibility of many crystalline phytochemicals including *β*-carotene and curcumin while providing improved* in vitro* anticancer activities. Additionally, many efforts have been devoted to the development and design of different nutraceutical delivery systems with significant progresses seen [136]. Besides that, nanoparticles generated using plant phytochemicals can be used in the discovery of biomarkers and refinement of diagnosis, thus forming the basis of new drugs for neurological disorders where new methods for delivery across the blood-brain barrier can be developed. Barriers to cancer fighting phytochemicals in various plant species and their future utility in the development of tumor-specific gold nanoparticles can provide remarkable opportunities towards better design and development of functional gold nanoparticles that can be safely synthesized and applied in oncological studies [137]. In addition, nanotechnology-based implants could facilitate the regeneration in the nervous system while femtolasers, nanorobots, and nanotechnology-derived devices will bring advancement in neurosurgery sector [138].

In summary, the factors that contribute to the successful nanotechnology-based phytochemicals delivery are improved solubility and bioavailability, less toxicity and side effects of phytochemicals, and enhanced biocompatibility. Future research efforts should focus on the development of new technologies for nanotoxicology, generation of the bases of nanobiomonitoring, and recognition of biological impacts of nanoparticles in the environment [139]. Based on these scenarios, it is an undeniable fact that the incorporation of phytochemicals and nanotechnology will be a new frontier in biomedicine field.

## 14. Conclusion

The present review focused on the anticancer activity of medicinal plants and the green synthesis of nanoparticles. Medicinal plants are the major sources of extremely active conventional drugs for the treatment of various types of disorders and diseases, including numerous forms of cancer. The active compounds isolated from medicinal plants may not specifically function as anticancer agents or drugs but may provide alternatives for the advancement of prospective cytotoxic agents. As research progresses, new technologies will aid in the improvement of the anticancer activities of drugs. Nanotechnology is a booming field related to nanoparticles, which have greater potential than normal-sized compounds. Metallic nanoparticles formed using plant extracts show enhanced tumor specificity, promising activity, and reduced toxic effects to healthy cells. The cytotoxic efficacy of nanoparticles is predominantly due to their large surface area, which enables efficient drug delivery, and some nanoparticles exhibit anticancer activity. However, most of the studies using medicinal plants and metallic nanoparticles have been conducted* in vitro*, and little* in vivo* data are available. Therefore, it is important to conduct research on medicinal plant extracts and metallic nanoparticles in* in vivo* models to extend these* in vitro* findings and to elucidate the mechanisms of action of active compounds and metallic nanoparticles for the advancement of anticancer drugs.

## Figures and Tables

**Table 1:** List of medicinal plants and phytochemicals and their anticancer activities.

Plant	Type of phytochemical(s)	Biological activity	References
*Alangium salviifolium*	Isoquinoline alkaloids and derivatives (IAD)	Ehrlich ascites carcinoma	[31]
*Aloe vera*	Aloin	Inhibition of human neuroectodermal tumors	[32]
*Azadirachta indica*	Limonoids	Murine Ehrlich carcinoma (EC) and B16 melanoma	[33]
*Apium graveolens*	Polyacetylenes	Leukemia cell lines	[34]
*Alisma orientale*	Triterpenes	HepG2, MDA-MB-231, and MCF-7 cell lines	[35]
*Alstonia yunnanensis*	IAD	Colon cancer	[36]
*Aristolochia cucurbitifolia*	IAD	Human liver cancer cell line	[37]
*Aristolochia manshuriensis*	IAD	Bone cancer	[37]
*Atractylodes macrocephala *	Sesquiterpenes	Lung carcinoma cells	[37]
*Berberis vulgaris*	Berberine	Breast, liver, and colon cancer cell lines (MCF-7, HepG2, and CACO-2)	[38]
*Brucea javanica*	Triterpenes	Bladder cancer	[38]
*Clausena harmandiana*	IAD	Cholangiocarcinoma	[39]
*Daphniphyllum glaucescens*	Terpenoids, alkaloids	General treatment of cancer	[40]
*Dictamnus dasycarpus*	Triterpenes	Human breast cancer cells	[41]
*Emblica officinalis*	Alkaloids	Antitumor activity	[42–44]
*Euphorbia fischeriana*	Diterpenes	General treatment of cancer	[45]
*Ginkgo biloba*	Terpenoids	Human breast cancer cell line	[46–48]
*Goniothalamus amuyon*	IAD	General treatment of cancer	[49]
*Gynura pseudochina (L.)*	Terpenoids, alkaloids	Breast cancer	[50]
*Hedyotis biflora*	Benzopyrones	General treatment of cancer	[51]
*Houpoea obovata*	Lignans	General treatment of cancer	[36]
*Ixeris chinensis*	Sesquiterpenes	General treatment of cancer	[52]
*Juglans mandshurica*	Quinones	Lung cancer	[53]
*Macleaya microcarpa*	IAD	General treatment of cancer	[54]
*Matricaria recutita*	Sesquiterpenes	Human HeLa cervix adenocarcinoma cells, K562 leukemia cells	[55]
*Nauclea orientalis *	IAD	Lung cancer	[56]
*Oroxylum indicum* *(L.) Kurz.*	Flavonoid	HeLa cells	[57]
*Petroselinum crispum*	Polyacetylenes	Leukemia cell lines	[34]
*Piper longum*	Amide alkaloids	HL60 and MCT-7 cell lines	[58, 59]
*Rhinacanthus nasutus*	Rhinacanthins	HeLaS3 cells	[60]
*Rubia cordifolia*	Quinones	P-388 cancerous cell line	[61–63]
*Schisandra henryi*	Triterpenes	Leukemia and HeLa cells	[64]
*Vitex rotundifolia*	Diterpenes	Leukemia/myeloma; colon cancer	[65, 66]
*Winchia calophylla *	Indole alkaloids and derivatives	P-388 and A-549 tumor cell lines	[67]
*Withania somnifera*	Alkaloids	Dalton's ascitic lymphoma	[68]

**Table 2:** The isolated compounds from different medicinal plants and their anticancer activities.

Medicinal plant name	Isolated compounds (structures are shown in Figure 1)	Anticancer activities	References
*Taxus baccata*	Cabazitaxel	Metastatic castration-resistant prostate cancer	[69]
*Colchium autumnale (autumn crocus)*	Colchicine	Multiple solid tumors (acts on matrix metalloproteases)	[70]
*Combretum caffrum*	Combretastatin	Human breast cancer	[71]
*Taxus* species	Docetaxol	Breast cancer; ovarian cancer; non-small-cell lung cancer (NSCLC)	[10]
*Glycyrrhiza uralensis*	Isoliquiritigenin	Human NSCLC; A549 lung cancer cell line	[72]
Needles of yew trees *Taxus baccata*	Larotaxel	Metastatic breast cancer; Bladder cancer; HSCLC; pancreatic cancer	[73] [74]
*Podophyllum peltatum* *Podophyllum emodi*	Podophyllotoxin	Lymphomas; bronchial and testicular cancers	[75]
*Taxus brevifolia*	Paclitaxel	Breast cancer; ovarian cancer; NSCLC	[10]
Polygonum roots, Peanut seeds, Berries and grapes	Resveratrol	Hepatoblastoma HepG2 and colorectal tumor SW480 cells	[76]
*Broussonetia papyrifera*	2S-abyssinone II		[77]
	Verubulin	Glioblastoma; brain tumors	[78–80]
*Catharanthus roseus* G. Don.	Vinblastine	Lymphocytic leukemia	[10]
*Catharanthus roseus* G. Don.	Vincristine	Lymphocytic leukemia	[10]
*Catharanthus roseus* (L.) G. Don (Apocynaceae)	Vindesine	Leukemias; lymphomas; advanced testicular cancer; breast and lung cancers; Kaposi's sarcoma	[10]
*Catharanthus roseus* (L.) G. Don (Apocynaceae)	Vinflunine	Leukemias; lymphomas; advanced testicular cancer; breast and lung cancers; Kaposi's sarcoma	[10]
Periwinkle plant (*Vinca* species)	Vinorelbine	Advanced breast cancer; advanced NSCLC	[81]

**Table 3:** Metallic nanoparticles at different sizes and shapes with anticancer activity.

Nanoparticles	Size (nm)	Shape	Cell line	Reference
Gold	2–16	Round	MCF-7 breast cancer cells	[82]
Chitosan	65	Round	MGC803 human gastric carcinoma cells	[83]
Gold	50	Rod	HeLa cells	[84]
Fe_3_O_4_	~5	Sphere	U-251 glioma cells	[85]
Fe_3_O_4_	~5	Sphere	T47D breast cancer cells	[85]
Folate-decorated quantum dots (QDs): loaded nanoparticles	280–300	Sphere	MCF-7 breast cancer cells and NIH-3T3 cells	[86]
Phosphatidylcholine-modified gold nanorods	65	Rod	HeLa cells	[87]
Highly water-dispersible and targeted CdS QDs	10–30	Sphere	CBRH7919 liver cancer cells	[88]
Solid lipid	145	Sphere	MCF-7 and MDAMB231 cells	[89]
Silver	16–20	Sphere	MCF-7 cells	[90]
